# Massively parallel sequencing of micro-manipulated cells targeting a comprehensive panel of disease-causing genes: A comparative evaluation of upstream whole-genome amplification methods

**DOI:** 10.1371/journal.pone.0196334

**Published:** 2018-04-26

**Authors:** Lieselot Deleye, Yannick Gansemans, Dieter De Coninck, Filip Van Nieuwerburgh, Dieter Deforce

**Affiliations:** Laboratory of Pharmaceutical Biotechnology, Ghent University, Ghent, Belgium; CNRS UMR7622 & University Paris 6 Pierre-et-Marie-Curie, FRANCE

## Abstract

Single Gene Disorders (SGD) are still routinely diagnosed using PCR-based assays that need to be developed and validated for each individual disease-specific gene fragment. The TruSight One sequencing panel currently covers 12 Mb of genomic content, including 4813 genes associated with a clinical phenotype. When only a limited number of cells are available, whole genome amplification (WGA) is required prior to DNA target capture techniques such as the TruSight One panel. In this study, we compared 4 different WGA methods in combination with the TruSight One sequencing panel to perform single nucleotide polymorphism (SNP) genotyping starting from 3 micro-manipulated cells. This setting simulates clinical settings such as day-5 blastocyst biopsy for Preimplantation Genetic Testing (PGT), liquid biopsy of circulating tumor cells (CTCs) and cancer-cell profiling. Bulk cell samples were processed alongside these WGA samples to serve as a performance reference. Target coverage, coverage uniformity and SNP calling accuracy obtained using any of the WGA, is inferior to the results obtained on bulk cell samples. However, results after REPLI-g come close. Compared to the other WGA methods, the method using REPLI-g WGA results in a better coverage of the targeted genomic regions with a more uniform read depth. Consequently, this method also results in a more accurate SNP calling and could be considered for clinical genotyping of a limited number of cells.

## Introduction

Several clinical settings such as PGT [1], liquid biopsy of CTCs and cancer-cell profiling of tiny biopsies could benefit from a method allowing genotyping of large panels of SNPs, starting with only a few cells as input. In this study, we simulate such a clinical setting by performing genotyping of a SNP panel on micromanipulated cells from a cell line. The study aims to evaluate the performance of hybridization-capture-based target enrichment sequencing after WGA on a few cells. More specifically, the study aims to provide performance data in a setting mimicking SGD diagnosis in PGT, empowering future clinical feasibility studies in the in-vitro fertilization (IVF) clinical practice.

Successful in-vitro fertilization may require preimplantation genetic testing (PGT) for the selection of an embryo free of chromosomal aberrations and single gene disorders (SGD) carried by its parents. Recently, shallow whole genome sequencing has proven to be useful for the genome-wide detection of chromosomal aberrations in PGT [2–4]. SGDs on the other hand, are still routinely diagnosed using PCR-based assays that are developed to target the specific gene fragment(s) involved in a single or a limited number of genetic diseases [5]. Development and validated application of these methods is time consuming. Established protocols often require adjustments for couples carrying slightly different mutations [6].

Whole Genome Amplification (WGA) can be used to amplify the DNA of the limited number of cells that can be biopsied from blastocysts for PGT. This pre-amplified DNA can be used to perform multiple PCR reactions, yielding multiple amplicons for the detection of disease-related mutations or insertions/deletions (indels). Massive parallel sequencing (MPS) can be used to sequence a pool of these different amplicons [7]. The cost and timescale are reduced by multiplexing different patient samples in a single sequencing run. Still, this approach requires a specific PCR primer design for each disease-associated locus. WGA combined with MPS could be used to sequence of the entire genome of the embryo, allowing simultaneous screening of Single Nucleotide Polymorphisms (SNPs) and chromosomal aberrations. Whole genome SNP analysis requires sequencing at high coverage for accurate detection, which currently is still too expensive for routine clinical applications.

The TruSight One Sequencing Panel (Illumina) targets 4,813 genes associated with known clinical phenotypes and enables simultaneous screening for mutations in these genes in a single sequencing run. Since only the genomic regions of interest are sequenced, a high depth is obtained for a relatively low cost. This panel was successfully applied to detect SGD during a prenatal screening using DNA extracted from amniotic fluid [8]. Because only 4–6 trophoblast cells can be biopsied in PGT, WGA is required to obtain enough DNA to start the TruSight One DNA capture. Unfortunately, WGA methods introduce representation bias and nucleotide changes during amplification. This hampers the applicability of these methods in PGT. Non-uniform amplification of regions across the genome may result in over- or underrepresentation of such genomic regions. Detection of mutations, more specifically SNPs, in insufficiently amplified (i.e. underrepresented) regions, can introduce genotyping errors. Additionally, the introduction of errors in the sequence during amplification could result in mutations being obscured or introduced. The commercially available WGA methods all have their specific strengths and weaknesses and choice is also influenced by the downstream application [9]. WGA methods based on multiple displacement amplification (MDA) are better suited for SNP detection because they use the high-fidelity phi29 polymerase. PCR-based WGA methods use DNA polymerases lacking proof-reading ability which introduce more errors, but yield a more balanced genomic amplification with less over- or underrepresented regions.

In this study, four different WGA methods were used to amplify DNA isolated from 3 micro-manipulated cells of the well-characterized and extensively sequenced NA12882 cell line of the Illumina Platinum Genome project. The currently most widely used WGA methods, two semi-random primer mediated PCR-based methods (SurePlex and MALBAC), a MDA method (REPLI-g) and a specific primer mediated PCR-based WGA method (Ampli1), were assessed with subsequent analysis using the TruSight One Sequencing Panel. The performance assessment is based on SNP calling accuracy, and on the number of allele dropouts. As many genetic conditions are multifactorial, and are influenced by multiple genetic variants and haplotypes, this assessment does not provide a performance analysis in terms of genetic diagnosis accuracy. A thorough clinical validation of this technique will be essential to reveal if WGA combined with target capture panels such as TruSight One can be used for clinical diagnosis.

## Material and methods

### Study design

**Fig 1:**
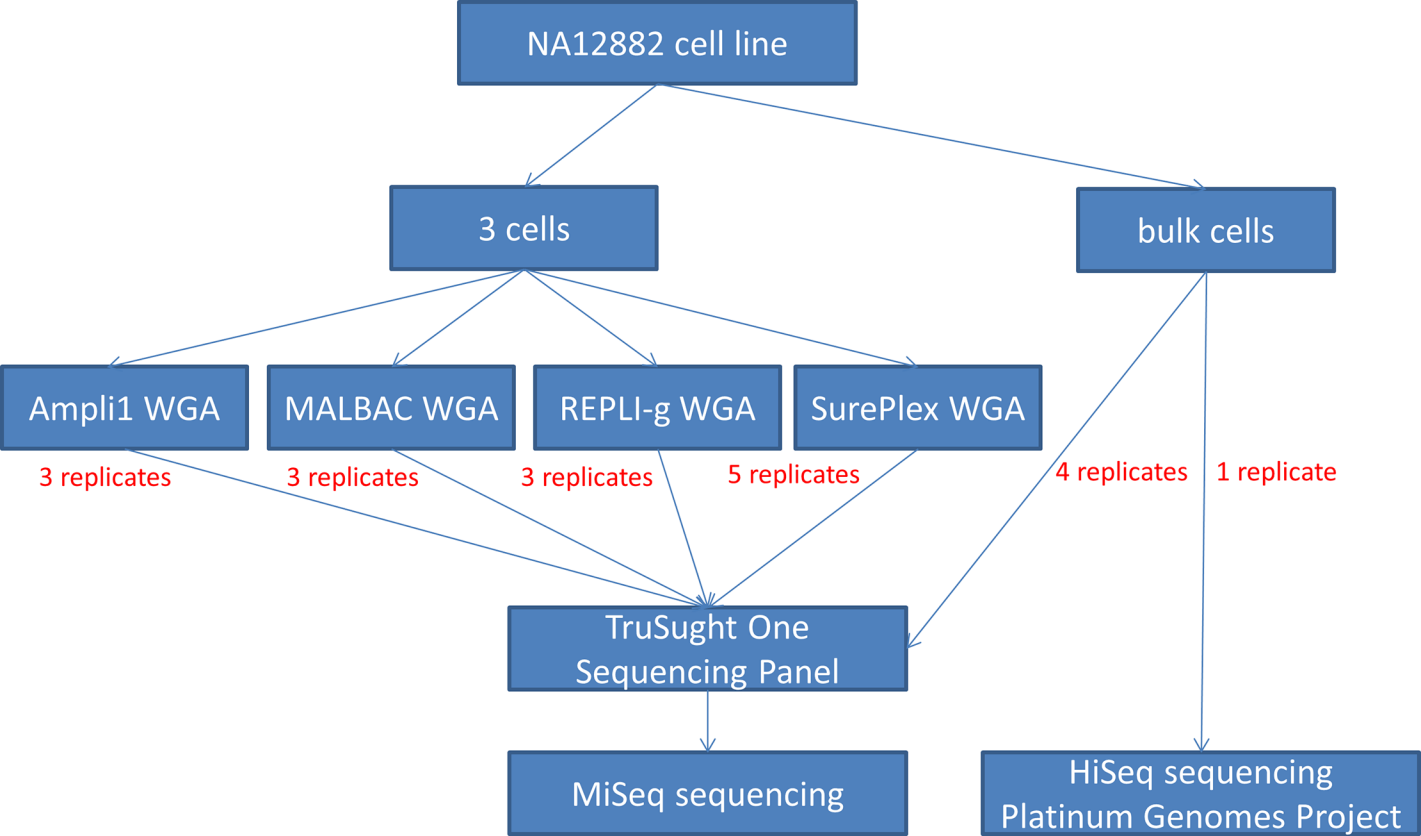
Experimental design. Three cell samples from the NA12882 cell line were amplified with either Ampli1 (3 replicates), REPLI-g (3 replicates), SurePlex (5 replicates) or MALBAC (3 replicates). Subsequently, samples were randomized for TruSight One capture in pools of 3 and those pools were sequenced on separate MiSeq runs. Four bulk DNA sample from the NA12882 cell line, not amplified before capture, were also randomly included in the pools.

This study was performed on cells from the NA12882 lymphatic male cell line (Coriell Institute, New Jersey, USA). The DNA of this cell line has been sequenced extensively (50X depth on a HiSeq2000) and the sequencing data is available online (http://www.illumina.com/platinumgenomes/). As this data allows for robust SNP detection, the NA12882 genome will be considered the true control reference in this study. We prepared 14 genomic DNA samples, each one starting from 3 NA12882 cells. These were amplified by 4 different WGA methods, resulting in 3 or 5 biological replicates per WGA method (5 for SurePlex, 3 for the others). Additionally, 4 genomic DNA samples were prepared from larger cell numbers and not submitted to WGA (further referred to as bulk DNA samples). The bulk DNA samples were included in the study to evaluate the TruSight kit performance in samples mimicking current clinical application of the kit [10]. Subsequent capture of gene regions with the TruSight One Sequencing panel (Illumina, San Diego, CA, USA) was performed on all samples (Fig 1). At the pooling step, four non-amplified bulk DNA samples and the WGA samples were randomly pooled per 3 to ascertain that variations observed after the capture step are caused either by the capture step or the preceding WGA. One MiSeq sequencing run was performed per pool of 3 samples.

### Growth and isolation of cells

The NA12882 cell line was grown in Roswell Park Memorial Institute (RPMI-1640) medium (Life technologies, Carlsbad, USA), supplemented with 15% fetal bovine serum (Life technologies, Carlsbad, USA) and 2mM L-Glutamine. For optimal growth, the cells were incubated at a temperature of 37°C and a 5% CO_2_ level. A known number of cells was isolated with an ergonomic denuding handle from STRIPPER (Origio, Måløv, Denmark) and MXL3-100 needles with a diameter of 100 μm (Origio, Måløv, Denmark). The desired number of cells was obtained by a serial dilution using spots of sterile phosphate buffered saline (PBS) (Life technologies, Carlsbad, USA) on a Petri dish (5.5 cm), performed under an Axiovert 25 light microscope (Zeiss, Jena, Germany). For optimal lysis, all cells were collected in a maximum volume of 1 μl. Immediately after collection, all samples were snap frozen in liquid nitrogen_._

For each bulk DNA sample, genomic DNA was extracted from 5x10^6^ cells using the DNeasy Blood & Tissue kit (Qiagen Hilden, Germany).

### Whole genome amplification

MALBAC (MALBAC kit, Yikon genomics YK001A/B version 1302.1, Jiangsu, China), SurePlex (Bluegnome, Cambridge, United Kingdom), REPLI-g (Qiagen, Hilden, Germany) and Ampli1 (Silicon Biosystems, Castel Maggiore, Italy) samples were processed following their respective kit manufacturer's instructions. A PBS blank and human positive control DNA sample was included for each method. All samples were purified using the Genomic DNA Clean & Concentrator kit (version 1.0.0, Zymo Research, Irvine, USA) according to the manufacturer's instructions with 5 X binding buffer. Concentration was measured using the Qubit dsDNA High Sensitivity Assay kit (Life technologies, Carlsbad, USA).

### Library preparation and sequencing

Sequencing libraries were prepared from 50 ng of the WGA-amplified or bulk DNA using the TruSight One Sequencing Panel (Illumina, San Diego, CA, USA) according to the manufacturer's instructions, except for the enrichment wash step where a thermocycler was used instead of the SciGene TruTemp heating system. Probe hybridization and capture was performed on equimolar pools of 3 samples and performed twice. The amount of sequence-able library fragments was determined by qPCR according to the Sequencing Library qPCR Quantification kit (Illumina, San Diego, USA). Finally, dual-index, paired-end 150 bp sequencing was performed on a MiSeq (Illumina). All sample pools were sequenced on separate MiSeq runs.

### Data analysis

Sequencing read quality was checked using FastQC (v0.11.5; http://www.bioinformatics.babraham.ac.uk/projects/fastqc/). Reads were aligned on the UCSC hg19 reference genome using bwa (v0.7.5) [11]. Mapping quality was evaluated using Qualimap (v2.2.1) [12]. Duplicate read marking and all bam file manipulations were done using Picardtools (v2.6.0; https://broadinstitute.github.io/picard/). Reads from the NA12882 Platinum Genome that aligned to the TruSight One Sequencing Panel regions were isolated using BEDTools (v2.26) [13]. SNP discovery in samples and the NA12882 reference was done using GATK (v3.6) [14] according to best practices instructions. Briefly, base quality scores were recalibrated in a two-pass covariation analysis supplemented with the dbSNP (release 135) [15] and Mills/1000 Genomes [16] data sets as a source of known variants. SNP calling was then performed at base resolution in discovery mode using an emission confidence threshold of 10 and a call confidence threshold of 30. The final SNP's were obtained by applying the following filter criteria to discard low quality SNPs: QD < 2.0, FS > 60.0, MQ < 40, MQRankSum < -12.5, ReadPosRankSum < -8.0. VCFtools (v0.1.13) [17] was used for all SNP comparisons between sample replicates and the NA12882 reference. Output file parsing, SNP counting and result table generation was done with python scripts.

### Sensitivity, false discovery rate (FDR)

Variants were compared between samples and the reference dataset for positions where both had sequencing data (comparable SNP’s). Variants not detected in either the sample or the reference dataset due to lack of sequencing data were counted and labeled as 'no data in sample' and ‘no data in reference’ respectively. The comparable SNP's were categorized as follows: True positives are defined as variant positions found in both the sample and the reference dataset. False positives are variant positions found in the sample but not in the reference dataset. Positions called as variants in the reference set but not in the sample are labeled false negatives. The sensitivity for SNP calling in a sample was defined as the number of true positives, divided by the sum of true positives and false positives. The FDR was defined as the number of false positives divided by the sum of true positives and false positives.

## Results

### Coverage and depth

To minimize run bias, samples amplified with the same WGA method were randomized over different MiSeq runs where possible (Table 1). The number of sequenced reads was variable between sample replicates. Two Ampli1 samples had a substantial lower yield than the other samples. This is probably caused by random errors during quantification and equimolar pooling of the sequencing libraries, combined with a variable cluster formation and detection across different sequencing runs. The percentage of reads aligning to the hg19 reference genome was similar for all samples including the bulk DNA samples (99.81±0.13%), as was the read alignment to the TruSight target regions (59.43±4.06%). This capture efficiency reflects the vendor’s performance specifications of the TruSight One kit. Mean depth across the targeted nucleotides correlated with the read yield and varied from 28X to 96X.

**Table 1:** Mapping statistics.

	*Sequencing run*	*Read count*	*Aligned reads (%)*	*Aligned reads on target (%)*	*Mean depth*	*Coverage uniformity (%)*	*Target coverage at 1X (%)*	*Target coverage at 10X (%)*	*Target coverage at 20X (%)*
*Bulk DNA-1*	1	14810732	99.82	60.91	78.85	95.14	99.60	97.38	92.95
*Bulk DNA-2*	2	15294678	99.92	63.12	83.20	94.82	99.62	97.22	93.07
*Bulk DNA-3*	3	19477275	99.86	58.25	95.86	94.75	99.62	97.51	94.43
*Bulk DNA-4*	3	18900774	99.85	59.48	94.65	94.48	99.60	97.36	94.10
*Ampli1-1*	4	12784447	99.89	57.82	66.23	49.82	75.63	53.00	43.93
*Ampli1-2*	2	5341335	99.94	66.62	31.01	50.22	65.96	43.59	33.15
*Ampli1-3*	1	4959973	99.84	62.27	27.48	47.75	63.29	39.59	29.27
*MALBAC-1*	5	14138090	99.36	52.02	61.03	47.92	78.59	51.27	39.77
*MALBAC-2*	6	11352373	99.78	61.06	58.21	46.91	78.90	49.68	38.30
*MALBAC-3*	5	14045595	99.81	53.72	62.62	46.86	78.76	51.39	40.13
*REPLI-g-1*	1	12359960	99.86	60.33	64.84	87.25	98.70	90.76	79.02
*REPLI-g-2*	2	13785283	99.92	59.22	71.01	89.10	98.99	92.84	83.18
*REPLI-g-3*	4	15937788	99.85	56.65	78.98	88.87	99.08	93.61	85.66
*SurePlex-1*	5	14277005	99.80	64.30	78.17	49.21	78.81	55.51	45.67
*SurePlex-2*	6	12662797	99.80	51.99	54.81	48.45	75.48	49.85	39.61
*SurePlex-3*	3	11649675	99.77	64.07	63.49	49.54	77.87	53.12	42.72
*SurePlex-4*	6	11670489	99.83	59.53	60.40	52.19	55.35	55.35	44.01
*SurePlex-5*	4	13676160	99.69	58.32	67.39	49.47	82.80	54.15	43.74

**Fig 2:**
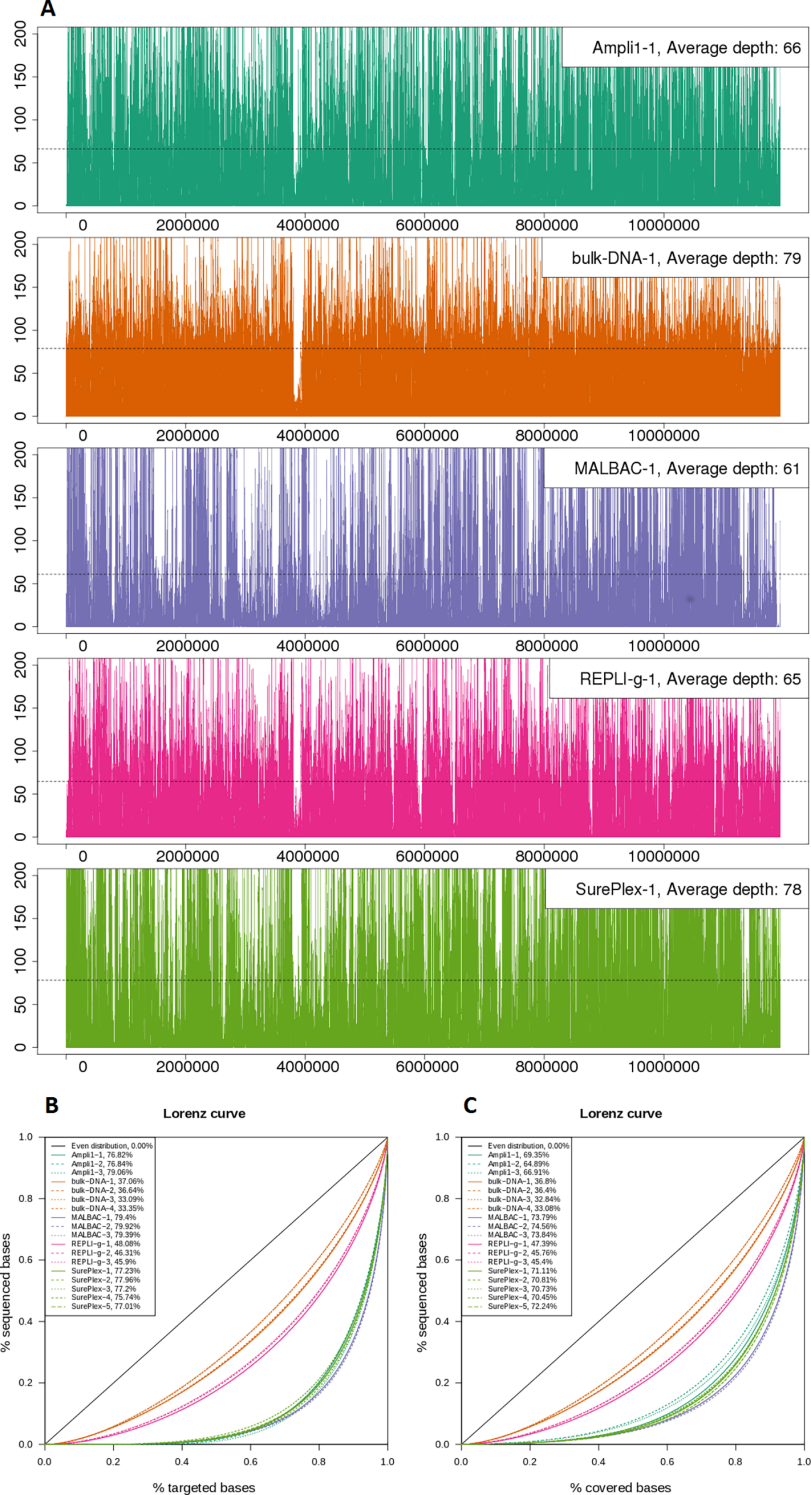
Read distribution. (A) Read depth calculated in 1 kb sliding windows across the concatenated target regions for one sample of each method. (B, C) Lorenz curves and Gini indexes describing how sequenced bases are distributed over targeted (B) and covered (C) bases.

The “coverage uniformity” (Table 1) is calculated as the percentage of targeted base positions for which the read depth is greater than 0.2 times the mean depth and thus is a measure for the number of bases that are not underrepresented in the read data. This coverage uniformity was different between the WGA methods, but similar for the repeats within a method. As expected, the coverage for the bulk DNA samples was most uniform (94.80±0.27%). REPLI-g amplified samples displayed a coverage uniformity closest to the bulk DNA samples (88.41±1.01%). The other WGA methods showed a lower uniformity: Ampli1, MALBAC and SurePlex have a uniformity of respectively 49.26±1.33%, 47.23±0.33% and 49.77±1.42%. Fig 2 visualizes this uneven distribution of reads over the targeted regions by means of different plots. Fig 2A plots the read depth across the targeted regions for one representative sample of each method. S1 Fig shows the same plots for the other replicate samples. The plot allows the visualization of larger regions with above or below average read depth. In terms of size of these alternating larger regions with high and low depth, there seems to be no pronounced difference between the different WGA methods. The over- and under-represented regions are not the same for the different methods, although there are some similarities: Bulk DNA, Ampli1, REPLI-g and SurePlex have a clear underrepresented region in common. Many of the over- and under-represented regions overlap between MALBAC and SurePlex. Fig 2B and 2C show Lorenz curves and Gini indexes describing how sequenced bases are distributed over targeted (B) and covered bases (C). The plots clearly show that the distribution of the reads from bulk DNA is closest to the even distribution. Comparing the WGA methods, the read distribution after REPLI-g WGA is closest to the even distribution.

**Fig 3:**
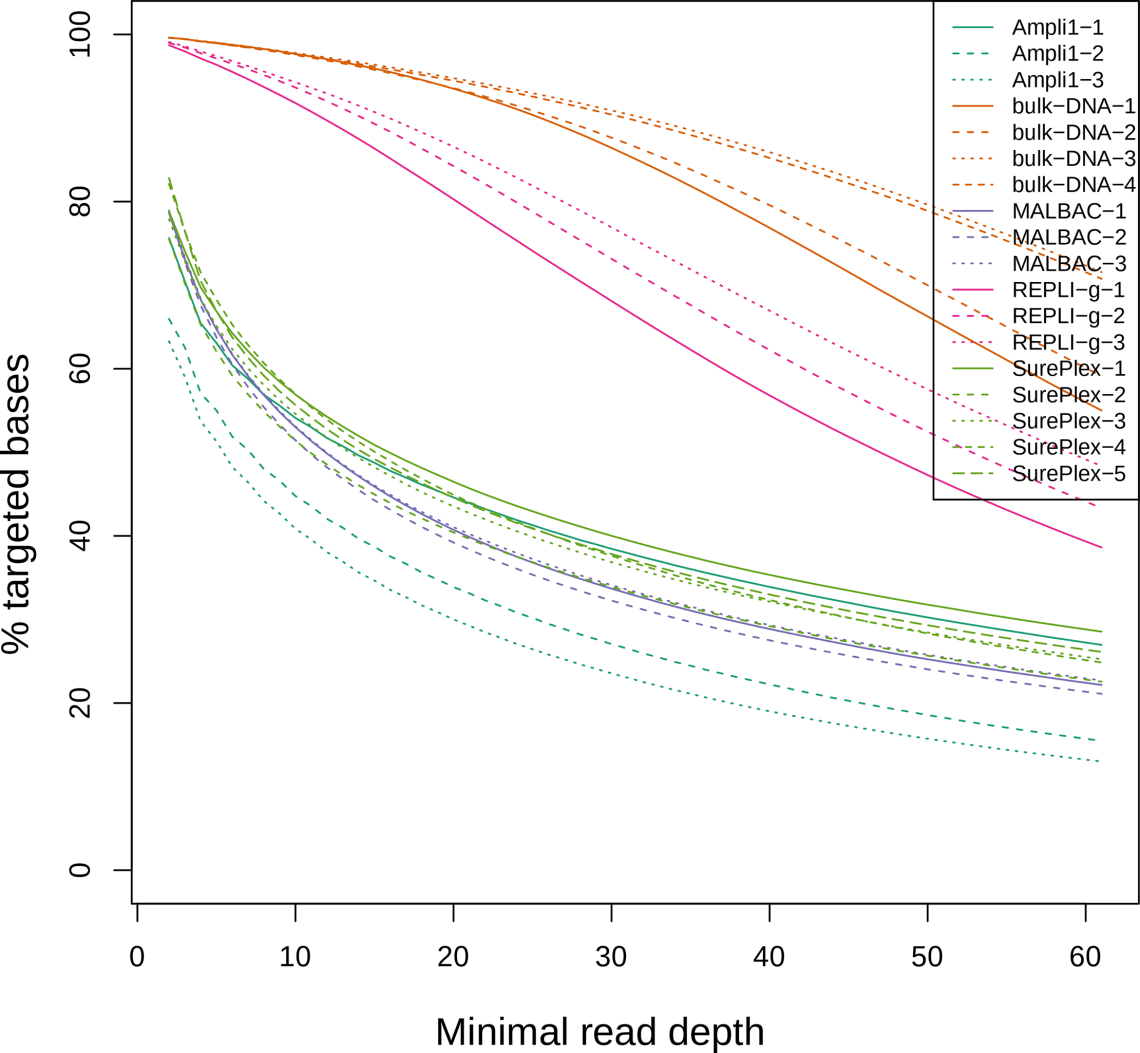
Percent target coverage at various minimum read depths.

Table 1 also shows the percentage of target regions covered with a minimal depth of 1X, 10X and 20X. For bulk DNA and REPLI-g samples, target region coverage with a minimal depth of 1X was almost complete (99.61±0.01% and 98.92±0.20% respectively). This percentage was at least 20% lower in Ampli1, MALBAC and SurePlex amplified samples. Considering regions covered with a minimal depth of 10X and 20X, the coverage decreases for all methods. For bulk DNA, the decrease is smaller compared to the WGA methods. For REPLI-g, the decrease is smaller compared to the other WGA methods. Fig 3 shows the percentage of targeted bases covered at a read depth between 1 and 60. Again, the plot shows that more targeted bases are covered at a higher depth when sequencing bulk DNA. Comparing the WGA methods, REPLI-g results in the highest percentage of targeted bases that are covered at higher depth. The plot also shows the decline in covered targeted bases when considering higher depths. This decline is less steep for bulk DNA and REPLI-g compared to the other WGA methods.

### SNP analysis

The online sequencing data for the Platinum Genome of the NA12882 cell line was mapped against the hg19 reference genome. An in-silico capture was performed on this mapping by isolating the reads aligning inside TruSight target regions. The SNPs called from this data are considered the reference set and were used to calculate the sensitivity and FDR for the samples (Table 2). The bulk DNA samples closely resembled the reference set, with a 96.8±0.2% sensitivity and a FDR of only 1.5±0.05%. This demonstrates the excellent performance of the capture kit when performed on unamplified DNA as specified in the kit’s manual. REPLI-g amplified samples, with a sensitivity of 93.3±1.5% and FDR of 3.0±1.2%, were the only WGA samples that performed nearly as good as the bulk DNA samples. All other WGA methods had a low sensitivity and a high FDR, indicating unreliable results. MALBAC amplified samples had the lowest sensitivity (65.7±14.3%), and the highest FDR (49.6±15.8%). Ampli1 and SurePlex were positioned somewhere in between. SurePlex had a sensitivity of 74.2±2.2% and an FDR of 36.8±8.2%, while Ampli1 had a comparable sensitivity of 73.9±0.5% but lower FDR of 20.7±4.2%. SNP analysis reflects the representation bias observed for the Ampli1, MALBAC and SurePlex samples. REPLI-g is the only WGA method producing SNP discovery results close to the bulk DNA samples.

**Table 2:** SNP analysis.

	True positives	False negatives	False positives	Sensitivity (%)	FDR (%)	No data in sample	No data in reference
Bulk DNA-1	7480	252	111	96.7	1.5	32	0
Bulk DNA-2	7477	256	108	96.7	1.4	31	0
Bulk DNA-3	7501	226	118	97.1	1.5	37	1
Bulk DNA-4	7479	253	116	96.7	1.5	32	0
Ampli1-1	4415	1517	1501	74.4	25.4	1832	0
Ampli1-2	3762	1327	904	73.9	19.4	2675	0
Ampli1-3	3642	1311	768	73.5	17.4	2811	0
MALBAC-1	3057	3169	6076	49.1	66.5	1538	2
MALBAC-2	4647	1624	2527	74.1	35.2	1493	0
MALBAC-3	4665	1659	4177	73.8	47.2	1440	0
REPLI-g-1	6980	643	325	91.6	4.4	141	4
REPLI-g-2	7199	452	202	94.1	2.7	113	3
REPLI-g-3	7229	438	150	94.3	2.0	97	1
SurePlex-1	4755	1528	3350	75.7	41.3	1481	0
SurePlex-2	4381	1711	3486	71.9	44.3	1672	0
SurePlex-3	4679	1556	2862	75.0	38.0	1529	0
SurePlex-4	4979	1524	1479	76.6	22.9	1261	0
SurePlex-5	4729	1850	2808	71.9	37.3	1185	0

The SNP calls were compared within the replicate samples for each WGA method. The 3 REPLI-g replicates had 6688 true positives and 67 false positives in common, comprising 93.7±1.80% and 32.8±12.05% respectively of the total number of true/false positives for each replicate. Of the common SNP calls, 99.01% are true positives, while 0.99% are false positives. These results were similar to those of the 4 bulk samples, in which 99.2% of the common SNP calls are true positives and 0.77% are false positives. This indicates that the detected false positives are not consistent between all replicates, but are introduced more randomly than true positives. True positives on the other hand, are highly consistent between these replicates. These results, as well as those for the other WGA methods are included in S1 Table.

### Allele dropout

Allele dropout (ADO), which occurs when one of the sample’s alleles is not present in sufficient quantities for amplification or detection, is a major concern for accurate genotyping. This results in missing data or spurious homozygotes and in a clinical context it could lead to misdiagnosis. In S2 Table, we calculated the maximum number of ADOs by summing following two situations that could have been caused by ADO: (1) SNP's that were not called in the samples due to lack of sequencing data (Table 2, 'No data in sample'). (2) SNP's that are heterozygous in the reference, but were called homozygous in the samples. As the latter situation could also have been caused by amplification or sequencing errors, the calculated number of ADOs is thus most probably an overestimation. The results show that the bulk DNA samples have an estimated maximum ADO level of 3.3 to 3.5%. For the WGA methods, the REPLI-g samples have the lowest ADO (6.7–10.2%). The samples from the MALBAC, Ampli1 and SurePlex WGA methods have ADO between 33.3 and 60.3%.

## Discussion

We performed a side-by-side SNP calling performance comparison of 4 widely used WGA methods in combination with hybridization-capture-based target enrichment sequencing. Performance of the same WGA methods for copy number aberrations (CNA) analyses has been studied extensively before [2,18–21]. SNP calling performance of these WGA methods in combination with target capture panels has also been studied [19,22,23]: Babayan *et al*. studied the accuracy of SNP/mutation, indel, and CNA calling after exome sequencing of Ampli1, REPLI-g, and PicoPlex WGA products [19]. Liu *et al*. performed variant calling on limited numbers of CTCs using GenomePlex and REPLI-g as the WGA methods and the GeneRead DNAseq Human CRC Panel as the gene panel [22]. SNP variants in CTCs have also been characterized using Ampli1 WGA and the Ion AmpliSeq Cancer Hotspot Panel v2 [23]. As WGA methods based on MDA are reportedly [9] better suited for SNP detection because they use the high-fidelity phi29 polymerase and amplify more regions of the genome compared to PCR-based methods, it is to be expected that REPLI-g would outperform MALBAC, SurePlex and Ampli1 in this study. Interestingly, a recently published paper by Borgström *et al*. [24] compares the performance of SurePlex, MALBAC, Ampli1 and REPLI-g Mini WGA methods for downstream exome capture sequencing starting from single cells. Although their experimental setup is similar to ours, these authors report that REPLI-g Mini performs worse than the other tested WGA methods. We can only speculate on the reason underlying these observations and the discrepancy with our results. Nevertheless, according to the kit manufacturer’s selection guide, the REPLI-g Mini kit might have been suboptimal for single cell WGA as it recommends starting from at least 10 ng of purified gDNA or from 300 cells. The REPLI-g Single Cell Kit used in our study allows starting from a single up to 1000 cells or from 10 pg to 10 ng purified gDNA, which in our 3-cell experiments may have provided us with more robust results.

## Conclusion

We compared the performance of 4 WGA methods to unamplified bulk DNA in combination with the Trusight One capture panel. Our study shows promising results when performing TruSight One after REPLI-g WGA on 3 cells: SNP calling sensitivity and FDR are similar, albeit inferior to those obtained using unamplified bulk DNA. Although a thorough clinical validation is needed to reveal if REPLI-g WGA combined with target capture panels such as TruSight One can be used for clinical diagnosis, our study shows a promising potential.

## Supporting information

S1 Fig Read distribution. Read depth calculated in 1 kb sliding windows across the concatenated target regions for replicate 2 and 3 of each method.
(TIF)

S1 Table SNP detection concordance between replicates per WGA method. (PDF)

S2 Table Allele dropout estimations. (PDF)
