# Supplementary figures and images for: Massively parallel sequencing of micro-manipulated cells targeting a comprehensive panel of disease-causing genes: A comparative evaluation of upstream whole-genome amplification methods
Source: PLoS One. 2018 Apr 26;13(4):e0196334. doi: 10.1371/journal.pone.0196334 (PMC5919401; doi:10.1371/journal.pone.0196334)

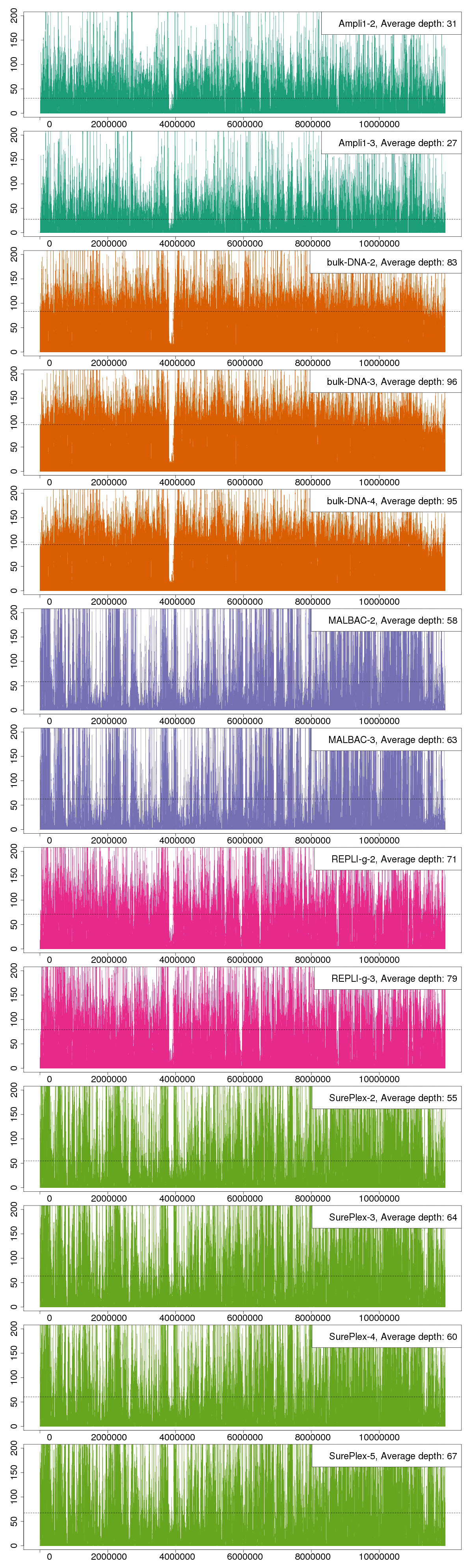

Supplement: S1 Fig — Read depth calculated in 1 kb sliding windows across the concatenated target regions for replicate 2 and 3 of each method. (TIF) [file pone.0196334.s001.tif]
