# Supplementary material for: Massively parallel sequencing of micro-manipulated cells targeting a comprehensive panel of disease-causing genes: A comparative evaluation of upstream whole-genome amplification methods
Source: PLoS One. 2018 Apr 26;13(4):e0196334. doi: 10.1371/journal.pone.0196334 (PMC5919401; doi:10.1371/journal.pone.0196334)
Supplement: S1 Table — (PDF) [file pone.0196334.s002.pdf]

S1 Table. SNP detection concordance between replicates per WGA method.

	Replicate	Number of SNP's	Common SNP's	% of total SNP's	Number of TP	Common TP	% of total TP	Number of FP	Common FP	% of total FP
Bulk DNA-1	1	7591	7383	97.3	7480	7326	97.9	111	57	51.4
Bulk DNA-2	2	7585	7383	97.3	7477	7326	98.0	108	57	52.8
Bulk DNA-3	3	7619	7383	96.9	7501	7326	97.7	118	57	48.3
Bulk DNA-4	4	7595	7383	97.2	7479	7326	98.0	116	57	49.1
Average±SD				97.2±0.19			97.9±0.14			50.4±2.07
Ampli1-1	1	5916	3053	51.6	4415	2979	67.5	1501	74	4.9
Ampli1-2	2	4666	3053	65.4	3762	2979	79.2	904	74	8.2
Ampli1-3	3	4410	3053	69.2	3642	2979	81.8	768	74	9.6
Average±SD				62.1±9.26			76.2±7.61			7.6±2.41
Malbac-1	1	9133	2339	25.6	3057	2316	75.8	6076	23	0.4
Malbac-2	2	7174	2339	32.6	4647	2316	49.8	2527	23	0.9
Malbac-3	3	8842	2339	26.5	4665	2316	49.6	4177	23	0.6
Average±SD				28.2±3.81			58.4±15.07			0.6±0.25
RepliG-1	1	7305	6755	92.5	6980	6688	95.8	325	67	20.6
RepliG-2	2	7401	6755	91.3	7199	6688	92.9	202	67	33.2
RepliG-3	3	7379	6755	91.5	7229	6688	92.5	150	67	44.7
Average±SD				91.8±0.64			93.7±1.80			32.8±12.05
SurePlex-1	1	8105	3393	41.9	4755	3376	71.0	3350	17	0.5
SurePlex-2	2	7867	3393	43.1	4381	3376	77.1	3486	17	0.5
SurePlex-3	3	7541	3393	45.0	4679	3376	72.2	2862	17	0.6
SurePlex-4	4	6458	3393	52.5	4979	3376	67.8	1479	17	1.1
SurePlex-5	5	7537	3393	45.0	4729	3376	71.4	2808	17	0.6
Average±SD				45.5±4.13			71.9±3.35			0.7±0.25
