# Supplementary material for: Massively parallel sequencing of micro-manipulated cells targeting a comprehensive panel of disease-causing genes: A comparative evaluation of upstream whole-genome amplification methods
Source: PLoS One. 2018 Apr 26;13(4):e0196334. doi: 10.1371/journal.pone.0196334 (PMC5919401; doi:10.1371/journal.pone.0196334)
Supplement: S2 Table — (PDF) [file pone.0196334.s003.pdf]

S2 Table. Allele dropout estimations. The maximum number of ADOs were calculated by summing following two situations that could have been caused by ADO: (1) SNP's that were not called in the samples due to lack of sequencing data (Table 2, 'No data in sample'). (2) SNP's that are heterozygous in the reference, but were called homozygous in the samples.

Sample	Total SNP in reference	Homozygous miscall	No data in sample	Estimated ADO count	Estimated ADO percent
Bulk DNA-1	7764	232	32	264	3.4
Bulk DNA-2	7764	238	31	269	3.5
Bulk DNA-3	7764	216	37	253	3.3
Bulk DNA-4	7764	231	32	263	3.4
Ampli1-1	7764	1513	1832	3345	43.1
Ampli1-2	7764	1258	2675	3933	50.7
Ampli1-3	7764	1216	2811	4027	51.9
Malbac-1	7764	3140	1538	4678	60.3
Malbac-2	7764	1486	1493	2979	38.4
Malbac-3	7764	1606	1440	3046	39.2
RepliG-1	7764	648	141	789	10.2
RepliG-2	7764	434	113	547	7.0
RepliG-3	7764	422	97	519	6.7
SurePlex-1	7764	1439	1481	2920	37.6
SurePlex-2	7764	1604	1672	3276	42.2
SurePlex-3	7764	1438	1529	2967	38.2
SurePlex-4	7764	1324	1261	2585	33.3
SurePlex-5	7764	1652	1185	2837	36.5
